# Dr. Grimes’ Address

**Published:** 1852-08

**Authors:** 


### DR. GRIMES' ADDRESS.

Dr. Samuel Grimes, of Delphi, Ind., delivered an excellent address before the Indiana Medical Convention, at New Albany, in May last, which has been published. We had the pleasure of listening to the address, and have since read it with much interest. We would be glad to copy it entire, but our limits will admit of only a few extracts.

"Before we blame with asperity the wrong bias of a portion of the community towards medical heresies and quackery, we, of the profession, ought to have the consciousness that we are clearly distinguishable from the whole tribe of pretenders, by our superior attainments, not only in medicine, but in general literature and science, as well as by our greater readiness of resource at a time of difficulty and danger in the sick room. The mere fact of our holding a diploma in Latin, while others put up with one in English, and of our believing in one doctrine, while they are clamorous in favor of another, will give us no valid claim on the world for higher professional rank, and greater skill in *practice*. We must rest our claims on something more substantive and appreciable than this. We may dwell with allowable pride on the antiquity, and learning, and signal philanthropy of the great family, with Hippocrates at its head, to which we *belong*. But the pride of ancestry becomes ridiculous if it is not sustained by contemporary merit. To be received in the great brotherhood and decorated with the order of merit, we ought to be able to show that we underwent early preparation by a good academic or collegiate education; and early training afterwards, by elementary medical instruction, in the office of an experienced physician, before we were enrolled and became regular and faithful attendants in a Medical School of repute and competent organization.

"Some, perhaps many of us, will confess, with regret, that we have not been able to comply with these requisites, in all their particulars. But, surely, this does not imply that we should withhold our advice and assistance, in order to remove the obstacles from the path of those coming after us, which embarrassed and impeded our own course. The times are more propitious to the zealous student now than they were in our early days; and, consequently, there is less excuse now than then for neglect, on the part of a parent, to procure for his son a good preparatory education, and on that of a physician to impart to his pupil suitable elementary instruction in medicine. Nor need any of us stand excused from the discharge of his duty to his pupil, if he received any such in his office, by the plea of his own defective attainments. However limited may be our reading and knowledge of medical literature, we can still point out to the young student a good book on each of the different branches of medicine; and early familiarize him with the qualities of drugs, and, to a certain extent, their pharmaceutical combinations.

"In pursuing the course here recommended, we shall discharge not only our duty to our students, but, also, to the several Medical Colleges to which they will resort after leaving us, for on the proper elementary instruction which these young men have received at our hands, will mainly depend their successful indoctrination in the higher principles and rational practice of Medicine by the professors in the schools. These gentletlemen are continually embarrassed in their teachings by not knowing what foundation has been laid by the private teachers, or by those who ought to have been such; nor how far they may take for granted an acquaintance with elementary truths on the part of the youthful auditors. One professor, for fear of going beyond the comprehension of his class, contents himself with uttering the merest *common places;* another aims to bring up his hearers to his own elevated standard. If it be said that the first is the most popular lecturer, does not the fact show the unpreparedness of the students for a course of instruction such as they ought to receive, and such as an able and conscientious professor ought to be able to give.

"The dignity, learning, elevated standing, and successful teachings of the Faculties of our Medical Colleges cannot be matters of indifference to us. They are, in one sense, our representatives, in another our auxiliaries; and whatever affects their reputation must re-act on the entire body of the profession. We cannot, therefore, be supposed to look with indifference on the organization of these colleges, nor be expected to give any countenance to those with the entire competency of whose Faculties we are not fully satisfied. A new Medical School cannot, like a new store, or a new manufactory, or a new line of stages, be patronized, merely because it is new, and to encourage trade and reduce prices, by competition. Nor can personal regards nor sectional pride furnish an apology for our sending our students, or otherwise giving our countenance to a school, formed in haste out of incongruous materials, and as if in a spirit of mere business speculation. The medical profession, at large, must divide the responsibility with the Medical Schools for the attainment and fitness of the graduates sent out annually from these latter, as candidates for practice and public patronage. If we send to the Medical Colleges crude materials, it cannot be expected that they should be at once fashioned into shape and harmonious proportions. If we send to them ignorant minds, we have no right to suppose that these will be instructed and taught the principles of medical logic, and philosophy in the course of a few months.

"I repeat, that we have a large share of responsibility for the manner in which Medical education is conducted. We send our students to be educated, we influence them in their selection of the school to which they go. We, therefore, mistake greatly our position, if we think that we can assume the office of judges and critics of Medical Schools without our participating in their cares and difficulties, and giving our counsel for their protection and guidance, when they come up to what we believe to be a good standard of teaching. The best assistance we can give them, is to send them students properly prepared to avail themselves of the higher course of instruction which the professors are ready to go through with. The best counsel we can offer to the professors, is for them to abide by their own convictions of duty in keeping up an elevated standard of medical attainments for their students, and taking adequate time for imparting their knowedge to these latter Not only are there more branches of medicine to be taught now, but each branch is much richer in details, than formerly; and hence it is clear that more time is required to teach them than formerly was requisite.

"It may be asked, whether we are ourselves, either by education or subsequent reading, competent judges, in the premises, of what is necessary on the score of Medical Education? It may be that many of us are not, in this sense *competent*, but the very consciousness of deficiencies, and experience of what we have suffered in consequence may naturally impel us to seek reform, and create a desire that others coming after us should enjoy advantages of which we were deprived.

"We may, however, all of us, in this age of periodical literature and abundant publications, readily procure the desired information on many subjects of immediate interest. The calls on our time and attention in the cares of practice, and especially country practice, must always prevent our being regular and systematic readers: but if we are bent on turning all the odd hours to account, and have a good medical journal, and the best monograph at our elbow, we shall be able to keep up, to a respectable extent, with the progress of our science, at least so far as to appreciate its extent and chief bearings, and the improvements in practice. In this way we can do justice to ourselves and to our students, and confer, if need be, understandingly with our brethren, the members of the Faculties of Medical Colleges."

